# Treatment of trauma-affected refugees with venlafaxine versus sertraline combined with psychotherapy - a randomised study

**DOI:** 10.1186/s12888-016-1081-5

**Published:** 2016-11-08

**Authors:** Charlotte Sonne, Jessica Carlsson, Per Bech, Ask Elklit, Erik Lykke Mortensen

**Affiliations:** 1 Competence Centre for Transcultural Psychiatry, Maglevaenget 2, 2750 Ballerup, Denmark; 2 Mental Health Centre North Zealand, University of Copenhagen, Dyrehavevej 48, 3400 Hillerød, Denmark; 3 National Center for Psychotraumatology, University of Southern Denmark, Campusvej 55, 5230 Odense, Denmark; 4 Institute of Public Health and Center for Healthy Aging, University of Copenhagen, Øster Farimagsgade 5, 1353 København K, Denmark

**Keywords:** Refugee, Trauma, Venlafaxine, Sertraline, Stress disorders, Post-traumatic stress disorders, Depression

## Abstract

**Background:**

The prevalence of trauma-related psychiatric disorders is high among refugees. Despite this, little is known about the effect of pharmacological treatment for this patient group. The objective of the present study was therefore to examine differences in the effects of venlafaxine and sertraline on Post-Traumatic Stress Disorder (PTSD), depression and functional impairment in trauma-affected refugees.

**Methods:**

The study was a randomised pragmatic trial comparing venlafaxine and sertraline in combination with psychotherapy and social counselling. PTSD symptoms were measured on the Harvard Trauma Questionnaire – part IV, which was the primary outcome measure. Other outcome measures included: Hopkins Symptom Check List-25 (depression and anxiety), Social Adjustment Scale – short version (social functioning), WHO-5 Well-being Index (quality of life), Crisis Support Scale (support from social network), Sheehan Disability Scale (disability in three areas of functioning), Hamilton Depression and Anxiety scale, the somatisation items of the Symptoms Checklist-90, Global Assessment of Functioning scales and the summarised score of pain in four body areas rated on visual analogue scales.

**Results:**

Two hundred seven adult refugee patients were included in the trial (98 in the venlafaxine and 109 in the sertraline group). Of these, 195 patients were eligible for intention-to-treat analyses. Small but significant pre-treatment to post-treatment differences were found on the Harvard Trauma Questionnaire and a number of other ratings in both groups. On the primary outcome measure, no difference was found in treatment effect between the sertraline and venlafaxine group. A significant group difference was found in favour of sertraline on the Sheehan Disability Scale.

**Conclusion:**

Sertraline had a slightly better outcome than venlafaxine on some of the secondary outcome measures, but not on the primary outcome measure. Furthermore, a higher percentage of dropouts was found in the venlafaxine group compared to the sertraline group. Although this could indicate that sertraline was better tolerated, which is supported by other studies, a final conclusion on tolerability cannot be drawn from the current study due to lack of systematic reporting of side effects.

**Trial Registration:**

ClinicalTrials.gov NCT01569685. Registration date: 28/2/12

**Electronic supplementary material:**

The online version of this article (doi:10.1186/s12888-016-1081-5) contains supplementary material, which is available to authorized users.

## Background

The prevalence of trauma-related psychiatric disorders such as Post-Traumatic Stress Disorder (PTSD) and depression is high among refugees [1]. Despite a rapidly increasing amount of studies on the treatment of PTSD in general, only few intervention studies have examined the pharmacological treatment of trauma-affected refugees [2, 3]. Therefore, clinical guidelines for the treatment of PTSD are based on reviews and meta-analyses, which mainly include studies on non-refugee PTSD populations [4–7]. However, the results from studies on non-refugee patients cannot be uncritically transferred to trauma-affected refugees due to a range of differences in biomedical and psychosocial profiles as well as trauma history [3, 8]. Survivors of civilian trauma have often been exposed to a single traumatic event, while refugees typically have a history of multiple and prolonged traumatisation [9]. Non-refugee PTSD patients also have the advantage of generally remaining in their habitual social, cultural and linguistic context after the traumatic event, which is, by definition, not the case for refugees. Consequently, trauma-affected refugees often present with a complex mixture of psychiatric symptoms that goes beyond merely PTSD in combination with psychosocial problems. Hence, they are often described among clinicians as a hard-to-treat patient group compared to other groups of PTSD patients [10].

Although most refugee mental health clinics offer combined treatment programmes including both pharmacological treatment and psychotherapy, the evidence for this approach is scarce. The single existent Cochrane review on combined pharmacotherapy and psychological treatment methods in patients with PTSD [6] identified only four studies that could be included in the review. These studies were all conducted on fairly small patient groups (the largest group had 65 participants for randomisation), and only one very small study (*n* = 10) included refugees [11]. The authors concluded that further research into the clinical management of PTSD was required, including trials with larger number of patients, using reliable and clinically meaningful outcome measurements such as remission of PTSD and functional outcomes. In addition, the authors called for studies of more homogeneous patient populations. While research on other groups of PTSD patients has burgeoned since, intervention studies concerning trauma-affected refugees are still limited in number and quality, and the vast majority are on psychological interventions only [12, 13].

Researchers at Competence Centre for Transcultural Psychiatry (CTP), where the present study was conducted, have prior to this study conducted research projects on the effects of combining medical and psychological treatment as well as the effects of different types of psychotherapy [14, 15]. In the present study, we therefore decided to investigate the pharmacological component of the standard combined treatment programme at CTP. When the present study was designed, the only official Danish guideline for the treatment of PTSD was the Danish Medical Technology report (MTV) “The treatment and rehabilitation of PTSD including traumatised refugees” from 2008 [16]. It concluded that selective serotonin reuptake inhibitors (SSRI), including the drug sertraline, were the best-documented pharmacological treatment of PTSD. However, both the MTV report and a Cochrane review on pharmacological treatment concluded that there is a need for more trials on patients with treatment refractory PTSD, such as that of many refugees, because SSRI seems to be inadequate for these patients [7, 16]. Studies of the pharmacological treatment of PTSD on other groups of trauma-affected patients have pointed towards the selective Serotonine-Noradrenaline reuptake inhibitor (SNRI) venlafaxine as an alternative treatment option [17]. Furthermore, SNRIs are known for their analgesic effect, which we hypothesised might be beneficial in a clinical population where most patients suffer from chronic pain [18].

To the best of our knowledge, only one study has investigated the use of venlafaxine in trauma-affected refugees [19]. This open-label study, with a total number of 32 participants (5 in venlafaxine treatment), compared sertraline, paroxetine, and venlafaxine. All 3 antidepressants produced statistically significant improvement by week 6 in PTSD symptom severity (PTSD Symptom Scale) while venlafaxine seemed to be less effective than the two other drugs in reducing symptoms of depression. However, the study had quite a few methodological problems (e.g. small groups sizes, gender imbalance among treatment groups, short study period, no intention-to-treat analyses), making results questionable.

### Aim of the study

As stated above, venlafaxine has shown promising results in non-refugee PTSD populations, but has only been compared to SSRIs in one small refugee study of limited quality. On this background, we found it appropriate to test the effects of venlafaxine versus an SSRI in a larger randomised trial in order to determine its effect in trauma-affected refugees. We therefore designed a randomised clinical trial comparing venlafaxine with sertraline, which was the standard choice of pharmacological treatment at CTP, where the study was conducted. The aim of the study was to examine differences in the effectiveness of venlafaxine and sertraline in reducing PTSD/depression symptoms and functional impairments in a sample of trauma-affected refugees referred to treatment at CTP. Based on the available literature on non-refugee PTSD patients, as well as the effect of SNRIs on general anxiety and depression, we hypothesised that venlafaxine would be as good as or better than sertraline in reducing PTSD and depression in our sample of trauma-affected refugees.

## Methods

The trial was a randomised 2-armed pragmatic trial using sertraline as an active control to venlafaxine. A combination of manualised psychotherapy and social counselling was used in both groups. The method is described thoroughly below. Additionally, a protocol paper has been published previously [20].

### Participants

CTP is a highly specialised transcultural psychiatric outpatient facility situated in the Capital Region of Denmark. The largest patient group is refugees with trauma-related mental health problems, but migrants with other mental health problems are also treated at the clinic. Participants in the present study were recruited from the total group of patients who had their first appointment at CTP between April 2012 and September 2013. Patients were invited to 1–3 h pre-treatment interviews with one of the clinic’s medical doctors in order to obtain psychiatric, medical, and trauma history, and to evaluate whether the patient belonged to the study target group and was motivated for treatment. If the patient fulfilled all inclusion but no exclusion criteria and gave written informed consent, the patient was included in the study and randomised to one of the two treatment groups. Inclusion criteria were: being a refugee or family-reunified to a refugee, being 18 years or above, having a history of at least one severe psychological trauma, fulfilling the diagnosis of PTSD and/or depression according to ICD-10 research criteria [21], being motivated for treatment, and giving informed consent to participate in the study. Exclusion criteria were: an ICD-10 F2x (schizophrenia, schizotypal and delusional disorders) or bipolar diagnosis, current abuse of drugs or alcohol, in need of acute admission to a psychiatric hospital, being pregnant or breastfeeding or being a woman in the reproductive age with a wish to conceive during the project period.

The diagnoses of PTSD, depression and enduring personality change after catastrophic experience were determined through a clinical interview by one of the CTP doctors who entered ICD-10 criteria for each of the diagnoses into a diagnostic algorithm. Psychotic and bipolar disorders were excluded using relevant parts of the SCAN interview and all doctors performing these interviews were certified SCAN raters. Trauma-related psychotic symptoms are relatively common in this patient group and were therefore not among the exclusion criteria [22, 23].

Patients were systematically inquired about their use of drugs and alcohol. Objective measures, such as an alcohol breath tester, were available but only used in cases where the clinician suspected that the patient was dishonest about current abuse.

Patients who fulfilled the inclusion criteria, but did not wish to participate in the study, were offered the clinic’s treatment as usual (TAU), which was similar to the treatment provided to the sertraline group. If patients did not want pharmacological treatment, it was still possible to receive treatment but, for obvious reasons, not possible to participate in the study.

**Fig. 1:**
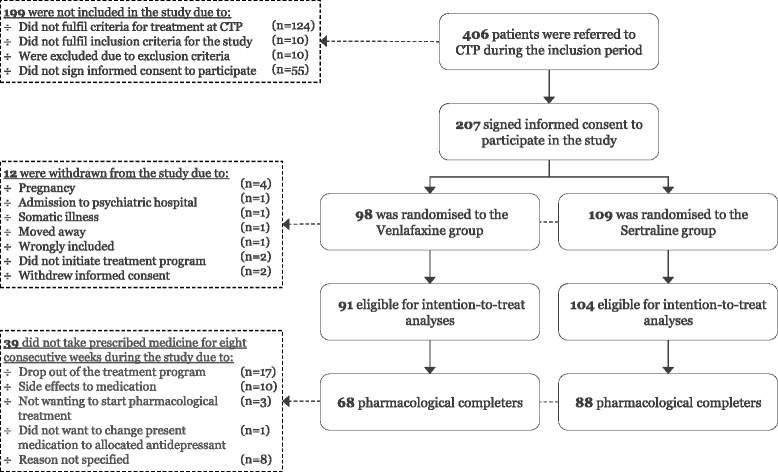
Participants flow through the trial

From 1 April 2012 until 15 September 2013, a total of 406 patients were screened for the trial, out of which 207 patients fulfilled all inclusion and no exlusion criteria and gave informed consent to participate in the study. The participants were randomised upon intake as described above – 98 to the venlafaxine group and 109 to the sertraline group. The last patient completed treatment in September 2014. Participants’ flow through the trial is depicted in Fig. 1.

### Randomisation and blinding

Randomisation by envelopes was performed, stratified by gender and level of severity of PTSD symptoms on the basis of Harvard Trauma Questionnaire (HTQ) score (above or below 3.2). A computer generated list was made by the Department of Biostatistics at the University of Copenhagen which was not otherwise involved in the trial. Consecutively numbered envelopes was sealed and these envelopes were administered by a group of secretaries at the central administration at Mental Health Centre Ballerup who had no other contact with the clinical staff at CTP during the study. Once a patient was included in the study, the doctor responsible for the inclusion phoned these secretaries and was informed of the group allocation.

Neither doctors nor patients were blinded in this study, while the raters administering the Hamilton Depression Scale (HAM-D) and the Hamilton Anxiety Scale (HAM-A) (please see below) were blinded to the time of the interview (so that the raters did not know whether it was a pre-treatment or post-treatment interview) and to the intervention group. A team of medical students, trained at CTP, administered the Hamilton scales. Inter-rater reliability was maximised by group ratings every 6–8 weeks.

### The intervention

The study compared a venlafaxine group with a sertraline group. For both groups, the treatment programme was planned to last 6–7 months. Patients in both groups were offered a total of 10 sessions with a psychiatrist/medical doctor (hereinafter referred to as doctor) and 16 sessions with a psychologist. All patients were offered one session with a social worker at the beginning and one at the end of the treatment programme, and more of such sessions were provided during the treatment programme if needed. Furthermore, it was optional for the patients to participate in group sessions with the social worker once a month. For all patients, the exact number of consultations as well as theme(s) of the session were registered at each consultation using a tick box sheet in accordance with the Treatment and Research Integrated Model (TRIM) used at CTP [24].

#### The venlafaxine group

Venlafaxine was given as slow-release tablets. The start dosage was 37.5–75 mg/day. For the first six weeks (phase 1), doctors aimed to see patients weekly and gradually increased the dosage by 37.5–75 mg at each consultation if tolerated by the patient. During phase 2 (the remaining part of the treatment programme), patients met with their doctor approximately once a month. If side effects did not prohibit, dosage was increased at these visits. The dosage could be increased up to the maximum recommended daily dosage of 375 mg/day.

#### The sertraline group

At the time of the study, sertraline was the first-line pharmacological treatment at CTP. The recommended start dosage was 25–50 mg. Like the venlafaxine group, patients in the sertraline group met with the doctor once a week during phase 1 and once a month during phase 2. Sertraline dosage was increased gradually by 25–50 mg per week for the first 6 weeks, and then adjusted once a month to a maximum of 200 mg/day.

Patients in both groups were instructed to take the prescribed medicine daily unless intolerable side effects occurred, in which case they should contact their doctor at CTP immediately. In addition to the trial medicine, all patients were offered supplementary treatment with mianserin if they suffered from severe sleep disturbances, which is the case for many patients with PTSD. Although no patients with a psychotic disorder were included in the study, some patients did suffer from trauma-related psychotic symptoms. If treatment with antipsychotic drugs was necessary from a clinical point of view, the patient would either continue with the antipsychotic treatment he/she was already receiving or start treatment with perphenazine. However, during the study period, perphenazine was taken off the Danish pharmaceutical market and we therefore decided to replace it with quetiapine in accordance with the current recommendation for first-line antipsychotic treatment in the clinical guidelines for the Capital Region of Denmark. However, CTP kept a stock of perphenazine for almost the entire remaining study period, and it is likely that only a very limited number of patients were affected by this change.

Whenever possible, patients were gradually taken off any other psychopharmacological treatments they received when entering the study. All these psychotropics were given free of charge by the clinic to the patient during the entire treatment programme regardless of whether they participated in the study or not.

### Psychotherapeutic treatment

Patients in both groups were offered 16 sessions of individual manual-based flexible cognitive behavioural therapy (CBT). The manual, developed by the psychologists employed at CTP, was based on the available literature and the experiences with the three previous manuals used during the prior randomised trials at the clinic [14, 15]. It set out flexible CBT, including elements from trauma-focused cognitive behavioural therapy (TF-CBT), acceptance and commitment therapy (ACT), stress management and mindfulness. All methods were adapted to fit the transcultural patient group [25].

### Compliance

Compliance is one of the major issues in most pharmaceutical studies with refugees and immigrants. Despite many attempts to secure compliance (written instructions in the patient’s own language, medical cards etc.), it was expected that many patients would have periods of poor compliance when, for example, running out of medicine, misunderstanding the dose or terminating pharmacological treatment without consulting the staff at the clinic.

Since frequent collection of blood samples could have a negative effect on the mental health of a patient group with a substantial amount of torture survivors, compliance was instead measured by pill count at each consultation with the doctor. If patients forgot to bring their medication to the consultation, this was noted in the patient file, and days of compliance were then calculated as the medical possession ratio (the number of days for which the patients had sufficient medicine) during the data management phase of the study. If patients had at least eight consecutive weeks of pharmacological compliance during the study period, they were defined as completers of the pharmacological part of the treatment programme.

### Measurements

The treatment outcome was measured by a combination of non-blinded self-report ratings and blinded observer ratings. The primary outcome measure was self-reported PTSD symptoms assessed using part IV of the Harvard Trauma Questionnaire (HTQ), which has been developed primarily for trauma-affected refugees and validated in several languages and settings [26, 27]. Secondary outcome measures included depression and anxiety symptoms measured on the Hopkins Symptom Check List-25 (HSCL-25) [28] and on the Hamilton Depression and Anxiety Ratings Scales (HAM-D and HAM-A) [29], social functioning measured on the Social Adjustment Scale Self Report (SAS-SR) short version [30], social support assessed on the Crisis Support Scale (CSS) [31], level of functioning assessed on the Sheehan Disability Scale (SDS) [32], quality of life assessed on the WHO-Five Well-being Index (WHO-5) [33], the somatisation scale of SCL-90, the mean score of pain in four different body areas rated on Visual Analogue Scales (VAS) and levels of symptoms and functioning assessed on the Global Assessment of Functioning (GAF) [34]. These measures were all self-report ratings except the GAF-scores, which were completed by the doctor in charge of the treatment and the HAM-D and HAM-A, which were completed by blinded assessors as described above. Based on our experience with the patient group, we estimated that the majority of the study participants would be out of job [14]. We therefore used a modified version of the SDS where the wording of the first item was ‘work/daily tasks’ (this version had been used in previous studies at CTP). For the same reason, we chose the SAS-SR over other rating scales of social function, since the phrasing of the questions similarly takes patients without jobs into consideration.

All self-report ratings were available in 5 languages: Danish, English, Farsi, Bosnian, and Arabic. Some of the ratings were available in Russian as well. Apart from SAS-SR short version and CSS, all outcome measures were used in previous randomised clinical trials at the clinic and were translated and implemented before this study commenced. In cases without a validated translation, a translated version was produced by standard translation and back-translation procedures. Whenever needed during ratings or treatment sessions, patients were assisted by a professional interpreter if they wished so.

Patients were asked to complete most self-report ratings three times during the study: at the pre-treatment interview (pre-treatment rating), right before starting psychotherapy, and at the end of the treatment programme (post-treatment rating). However, as a relatively high number of patients did not complete the second rating before starting psychotherapy, only the pre- and post-treatment ratings were used in the analyses (please see the data management section below).

If the pre-treatment interview was conducted more than two months before the patient’s first consultation with a doctor, a new rating was conducted at the first treatment session with the doctor, and this new rating was then used as the pre-treatment rating. SAS-SR and CSS were completed twice during the study: at the first and at the last consultation with the social worker. The blinded Hamilton interviews were also carried out twice: at the beginning and at the end of the treatment programme. Patients who terminated the treatment programme before time were encouraged to complete the same post-treatment questionnaires as those who completed the full treatment programme.

#### Reliability

Reliability of the ratings used in the study was generally high. Cronbach’s alpha was calculated for all ratings that included more than one item and ranged between 0.69 (CSS) and 0.90 (HSCL-25). For the primary outcome measure, the HTQ, Cronbach’s alpha was 0.79.

### Data management and statistics

Data was entered into the clinic’s Access database twice and discrepancies corrected according to the case record files. Rating scale scores were recorded as missing if more than half of the scale items were unanswered. Analyses were performed using Stata 14.

Pre-treatment data and descriptive data on the treatment provided were analysed for group differences using chi-square and t tests. Cronbach’s alpha was determined for all pre-treatment ratings. Differences between post-treatment and pre-treatment ratings were analysed using a mixed model, which for each outcome included intervention group, rating time (pre-treatment vs. post-treatment) and the interaction between intervention group and time as predictors. Using Stata’s “margins’ and “contrast” commands, the model made it possible to estimate pre- and post-treatment group means, to test group differences in pre- and post-treatment ratings separately; differences between pre- and post-treatment ratings in each group; and group differences in differences between pre- and post-treatment ratings (corresponding to the interaction between intervention group and rating time). This analysis was conducted on both a reduced sample, consisting of patients with complete data on both pre- and post-treatment ratings, and on an intention-to-treat sample, which included all patients with pre-treatment ratings. Pre-post-treatment differences are often correlated with pre-treatment scores, and, consequently, differences between the sertraline and venlafaxine groups were also analysed with regression models that included the pre-treatment scores on each rating scale and an indicator variable for treatment group as predictor and the score on the rating scale as outcome. To conduct intention-to-treat analyses, the regression analyses were conducted using Full Information Maximum Likelihood (FIML), which incorporates all available information including pre-treatment scores for patients without post-treatment scores. The structural equation modeling procedure “sem” in Stata 14 was used to conduct these analyses. Both the mixed model and the regression models were conducted with robust standard errors.

## Results

### Characteristics of participants

Pre-treatment characteristics for the two groups, including sociodemographic data, trauma history, and psychiatric diagnoses, are illustrated in Table 1. Almost 99 % of the patients had comorbid depression and a little more than 40 % were diagnosed with enduring personality change after catastrophic experience (F.62.0). Another comorbid psychiatric disorder was registered for almost 12 %, but this disorder was not neccarily specified in accordance with ICD-10 in the patient files. The vast majority of the diagnoses were anxiety disorders, with generalised anxiety (F.41.1) being the most frequent. None of the analysed variables differed significantly between groups, although there was a borderline significant difference in distribution of torture survivors (*p* = 0.054). Just over 60 % of the patients required an interpreter for treatment sessions (61 % for medical doctor sessions and 63 % for psychologist sessions).

**Table 1:** Pre-treatment characteristics for the study population

Pre-treatment characteristics	All (*n* = 207)^a^	Venlafaxine (*n* = 98)^a^	Sertraline (*n* = 109)^a^
N(%)			
Demographic information			
Male gender	124 (60.2)	61 (62.2)	63 (58.3)
Country of origin			
Ex-Yugoslavia	20 (9.7)	11 (11.2)	9 (8.3)
Iran	28 (13.6)	13 (13.3)	15 (13.9)
Iraq	71 (34.5)	34 (34.7)	37 (34.3)
Afghanistan	28 (13.6)	10 (10.2)	18 (16.7)
Lebanon	26 (12.6)	12 (12.2)	14 (13.0)
Other	33 (16.2)	18 (18.4)	15 (13.9)
Diagnosis (ICD-10) in addition to PTSD			
Depression	204 (98.6)	96 (97.96)	108 (99.08)
Enduring personality change after catastrophic experience (F.62.0)	80 (40.8)	38 (41.30)	42 (40.38)
Other psychiatric disorder	24 (11.7)	12 (12.24)	12 (11.11)
Trauma history			
Imprisonment	110 (53.4)	57 (58.8)	53 (48.6)
Torture**	99 (48.1)	54 (55.1)	45 (41.7)
Refugee camp	52 (25.7)	22 (22.9)	30 (27.5)
Psychosocial status			
Education >10 years from home country	98 (50.8)	50 (53.2)	48 (48.5)
Presently employed/studying	14 (7.0)	7 (7.3)	7 (6.8)
Living alone all the time	51 (25.8)	21 (21.9)	30 (29.4)
Have got children less than 18 years old	137 (68.8)	68 (70.8)	69 (67.0)
Mean(SD)			
Age	43.7 (9.7)	43.2 (9.6)	44.0 (9.7)
Years since arrival in Denmark	14.6 (7.3)	14.1 (7.1)	15.1 (7.4)

Pre-treatment characteristics for the venlafaxine and sertraline groups

*N* Number of study participants, *SD* Standard deviation

** Group difference borderline significant, *p* = 0.054

^a^Not all variables available for the entire patient group

### Attrition and compliance with treatment programme

Twelve patients (seven patients in the venlafaxine group and five in the sertraline group) were excluded from the study: four due to pregnancy, two due to hospital admissions (one to a psychiatric and one to a somatic ward), one was wrongly included in the study (was not a refugee), one moved to another part of Denmark, two changed their minds about initiating treatment at CTP and two withdrew informed consent. Accordingly, 195 patients (94.2 %) were available for intention-to-treat analyses.

A total group of 156 patients (75.4 %) completed minimum 8 weeks of pharmacological treatment in accordance with the group to which they were randomised (68 in the venlafaxine group and 88 in the sertraline group). The majority of the non-completers took the allocated antidepressants during part of the treatment programme, but either stopped before 8 weeks or took their medication irregularly during large parts of the treatment programme. Reasons reported for not completing 8 weeks of pharmacological treatment included dropping out of the CTP treatment programme (*n* = 17), side effects to medication (*n* = 10), not wanting pharmacological treatment after all (*n* = 3) or not wanting to change present medication to allocated antidepressant (*n* = 1). No reasons were reported for the remaining non-completers (*n* = 8).

### Pharmacological side effects

As mild side effects are common for both venlafaxine and sertraline, it was expected that many patients would have side effects during parts of the study, which was also the case according to the clinicians’ reports. Changes in the patients’ physical condition were registered at each medical doctor session, but only unexpected or serious events/side effects were systematically collected and reported to the ethics committee and the Danish Medicines Agency, in accordance with Danish legislation at the time being.

Only 10 patients terminated their pharmacological treatment because of reported side effects. However, as 17 patients did drop out of the entire treatment programme without a specified reason and no reasons were reported for an additional eight non-completers, the actual number of patients with intolerable side effects could have been higher. Three patients had to switch drugs during the trial: two did not tolerate venlafaxine but did tolerate sertraline and one only tolerated venlafaxine. These three patients were kept in the group to which they were randomised during the intention-to-treat analyses.

### Treatment

Patients received a mean number of eight medical doctor sessions, 10 psychologist sessions and two social worker sessions with no significant differences between the two groups. Only 29 patients (14 in the venlafaxine group and 15 in the sertraline group) participated in at least one group session with a social worker.

Mean treatment length was 6.3 months. Mean dose of sertraline was 96.21 mg and mean dose of venlafaxine was 125.41 mg. Ten patients in the venlafaxine group received a mean dose larger than 225 mg. A total number of 63 patients (69.23 %) in the venlafaxine group and 80 patients (76.92 %) in the sertraline group received add-on mianserin at some point during the study, with a mean dose of 13.57 mg (no significant group difference). A total of 19 patients in the venlafaxine group (20.88 %) and 23 patients in the sertraline group (22.12 %) received treatment with antipsychotics at some point, although many of these were phased out during the study.

### Outcomes

#### Pre-treatment and post-treatment ratings

The primary outcome measure HTQ was completed both at pre-treatment and post-treatment by 154 patients. With regard to the other ratings, the number of patients who had completed both pre-treatment and post-treatment ratings ranged between 123 (GAF-F) and 158 (HAM-D).

Table 2 presents the results of the mixed model intention-to-treat analysis (the results of the reduced sample are presented in a Additional file 1: Table S1). For the pre-treatment ratings, the two groups only differed significantly on the SDS score and there were no significant group differences on the post-treatment scores. The table also shows the differences between pre-treatment and post-treatment ratings for each group and the group differences with respect to the difference between pre- and post-treatment ratings.

**Table 2:** Score differences between pre-treatment and post-treatment ratings

Rating	Groups and differences	Mean pre-treatment score (SE)	Mean post-treatment score (SE)	Difference (SE)	*P*-value	Effect size
Symptoms self-ratings						
HTQ	Sertraline	3.24 (0.04)	3.02 (0.06)	**0.22 (0.06)**	<0.01**	**0.54**
	Venlafaxine	3.18 (0.05)	3.05 (0.06)	**0.13 (0.06)**	0.02*	**0.32**
	Difference	0.06 (0.06)	−0.03 (0.09)	0.09 (0.08)	0.27	0.22
HSCL-25	Sertraline	3.03 (0. 05)	2.85 (0.07)	**0.18 (0.07)**	<0.01**	**0.39**
	Venlafaxine	3.04 (0.05)	2.94 (0.06)	**0.10 (0.05)**	0.05*	**0.22**
	Difference	−0.01 (0.07)	−0.09 (0.09)	0.08 (0.09)	0.37	0.17
SCL-90	Sertraline	2.40 (0.08)	2.35 (0.10)	**0.05 (0.10)**	0.64	**0.06**
	Venlafaxine	2.50 (0.08)	2.53 (0.09)	*−0.03 (0.07)*	0.69	*0.04*
	Difference	−0.10 (0.10)	−0.18 (0.13)	0.08 (0.12)	0.54	0.10
VAS	Sertraline	6.38 (0.24)	6.17 (0.28)	**0.21 (0.26**)	0.43	**0.26**
	Venlafaxine	6.61 (0.24)	6.68 (0.24)	*−0.07 (0.20)*	0.72	*0.03*
	Difference	−0.23 (0.34)	−0.51 (0.36)	0.28 (0.33)	0.40	0.12
Life quality/level of functioning self-ratings						
WHO-5	Sertraline	12.73 (1.36)	22.21 (2.67)	**−9.48 (2.42)**	<0.01**	**0.65**
	Venlafaxine	15.00 (1.64)	17.75 (2.24)	**−2.75 (2.16)**	0.20	**0.19**
	Difference	−2.27 (2.13)	4.46 (3.49)	6.73 (3.24)	0.04*	0.47
SDS	Sertraline	24.65 (0.53)	21.81 (0.88)	**2.84 (0.85)**	<0.01**	**0.48**
	Venlafaxine	22.71 (0.69)	23.20 (0.79)	*−0.49 (0.86)*	0.57	*0.08*
	Difference	1.93 (0.87)*	−1.39 (1.17)	3.32 (1.21)	<0.01**	0.56
SAS-SR	Sertraline	2.93. (0.07)	2.68 (0.08)	**0.25 (0.07)**	<0.01**	**0.36**
	Venlafaxine	2.96 (0.07)	2.80 (0.0.08)	**0.16 (0.08)**	0.04*	**0.23**
	Difference	−0.03 (0.10)	−0.12 (0.11)	0.09 (0.11)	0.39	0.13
CSS	Sertraline	22.34 (0.89)	22.89 (0.80)	**−0.55 (0.79)**	0.48	**0.07**
	Venlafaxine	22.14 (0.76)	22.40 (0.79)	**−0.26 (0.73)**	0.72	**0.03**
	Difference	0.20 (1.17)	0.49 (1.13)	0.29 (1.07)	0.79	0.04
Observer ratings						
HAM-D	Sertraline	23.69 (0.55)	22.33 (0.85)	**1.36 (0.79)**	0.08	**0.24**
	Venlafaxine	23.69 (0.62)	22.46 (0.89)	**1.23 (0.82)**	0.13	**0.22**
	Difference	0.00 (0.83)	−0.13 (1.23)	0.13 (1.14)	0.91	0.02
HAM-A	Sertraline	26.74 (0.68)	26.41 (1.04)	**0.33 (0.97)**	0.73	**0.05**
	Venlafaxine	27.14 (0.72)	26.05 (1.05)	**1.09 (1.00)**	0.28	**0.16**
	Difference	−0.40 (0.99)	0.36 (1.49)	−0.76 (1.39)	0.58	0.11
GAF-S	Sertraline	47.43 (0.57)	51.33 (0.93)	**−3.90 (0.79)**	<0.01**	**0.68**
	Venlafaxine	48.14 (0.61)	51.82 (0.94)	**−3.68 (1.03)**	<0.01**	**0.64**
	Difference	−0.70 (0.83)	−0.48 (1.32)	0.22 (1.29)	0.86	0.04
GAF-F	Sertraline	48.37 (0.68)	50.28 (0.92)	**−1.91 (0.79)**	0.02*	**0.29**
	Venlafaxine	49.04 (0.68)	51.91 (0.96)	**−2.87 (0.89)**	<0.01**	**0.43**
	Difference	−0.67 (0.97)	−1.63 (1.33)	0.96 (1.19)	0.42	0.06

HAM = 0–4 (0 best score), GAF = 0–100 (100 best score), WHO-5 = 0–100 (100 best score), SDS = 0–30 (0 best score), SAS-SR = 1–5 (1 best score), CSS = 1–7 (7 best), HTQ, HSCL-25, SCL = 1–4 (1 best score), VAS = 0–10 (0 best score)

*WHO-5* WHO-Five Well-being Index, *SDS* Sheehan Disability scale, *SAS-SR* Social Adjustment Scale - Self Report, *CSS* Crisis Support Scale, *HTQ* Harvard Trauma Questionnaire, *HSCL-25* Hopkins Symptom Checklist-25, *SCL-90* Symptom Checklist-90, *VAS* Visual Analogue Scale (for pain), *HAM-A* Hamilton Anxiety scale and *HAM-D* Hamilton Depression scale, *GAF* Global Assessment of Functioning (S = symptom score and F = functioning score)

Overview over pre-treatment and post-treatment rating scores for the intention-to-treat sample. In the right column the *p*-values refer to the significance of differences between pre- and post-treatment ratings in each group and the significance of group differences in the difference between pre- and post treatment ratings (corresponding to the interaction between intervention group and rating time). Based on the mixed model for the full sample group differences at the pre- and post-treatment assessments were estimated as simple main effects and so were the pre-post treatment differences for each intervention group. The group difference in pre-post treatment differences corresponds to the interaction coefficient in mixed model. SE = Robust standard error Effect size: The effect size calculated as the pre-post score difference/the baseline standard deviation

* = statistically significant (*p* = 0.05 or below), ** = higly statistical significant (*p* = 0.01 or below)

**Bold =** Improvement, *Italic* = Deterioration

The table shows small but significant improvements in both groups on the primary outcome measure HTQ as well as on a number of other ratings: HSCL-25, SAS-SR, GAF-S and GAF-F. On the blinded Hamilton ratings, the CSS, the SCL-90 and the VAS pain scale, no significant changes were found in either of the groups. On the WHO-5, we found a significant improvement in the sertraline group only, and the difference between the two groups was reflected in a significant interaction between group and rating time (*p* = 0.04). This pattern was even clearer on the Sheehan Disability Scale, where we found a significant improvement in the sertraline group, but a non-significant deterioration in the venlafaxine group. This group difference was reflected in a significant interaction between group and rating time (*p* < 0.01).

#### Differences in effects between groups

Table 3 illustrates the differences between the sertraline group and the venlafaxine group in the intention-to-treat sample analysed with Full Information Maximum Likelihood, which means that we were able to use data from all 195 patients included in the intention-to-treat sample. No significant treatment difference was found on the primary outcome measures. On the other outcome measures, we found a significant group difference on SDS only and borderline significant differences between the treatment groups on WHO-5 (*p* = 0.07). These group differences were both in favour of sertraline and in line with the results presented in Table 2.

**Table 3:** Regression coefficients for group differences at follow up

Rating	Regression coeffiecient, B (95 % CI)	Beta-cofficient	SE	Z	*P*
Adjusted for pre-treatment rating scores					
HTQ	**0.07 (−0.09 – 0.22)**	**0.06**	0.08	0.84	0.40
HSCL-25	**0.07 (−0.10 – 0.23)**	**0.05**	0.08	0.80	0.42
SCL	**0.12 (−0.12 – 0.35)**	**0.07**	0.12	1.03	0.31
SDS	**2.31 (0.10 – 4.52)**	**0.16**	1.13	2.05	0.04*
WHO-5	**−5.79 (−12.05 – 0.46)**	**−0.13**	3.19	−1.82	0.07*
VAS	**0.36 (−0.23 – 0.96)**	**0.08**	0.30	1.20	0.23
SAS-SR	**0.10 (−0.09 – 0.29)**	**0.07**	0.10	1.06	0.29
CSS	**−0.35 (−2.18 - 1.47)**	**−0.02**	0.93	−0.35	0.71
GAF-F	*1.22 (−1.03 – 3.47)*	*0.08*	1.15	1.07	0.29
GAF-S	*0.06 (−2.37 – 2.48)*	0.00	1.24	0.04	0.96
HAM-D	**0.19 (−1.94 – 2.33)**	**0.01**	1.09	0.18	0.86
HAM-A	*−0.57 (−3.19 – 2.04)*	*−0.03*	1.34	−0.43	0.67

Post-treatment differences between the venlafaxine and sertraline groups for the intention-to-treat sample

*CSS* Crisis Support Scale, *GAF* Global Assessment of Functioning, *HAM-A* Hamilton Anxiety scale, *HAM-D* Hamilton Depression scale, *HTQ* Harvard Trauma Questionnaire, *HSCL-25* Hopkins Symptom Checklist-25, *SAS-SR* Social Adjustment Scale - Self Report, *SCL-90* Symptom Checklist-90, *SDS* Sheehan Disability scale, *VAS* Visual Analogue Scale, *WHO-5* WHO-Five Well-being Index, *B* Regression coeffiecient, *CI* Confidence interval, *Z* Z-value *SE* Robust standard error of the regression coeffiecient, B, *P P*-value for the regression coeffiecient, B

**Bold**: In favor of sertraline

*Italic*: In favor of venlafaxine

**Bold** and *: Statistically significant

*: Marginally significant

## Discussion

Our study is the largest study ever comparing different pharmacological treatment options for trauma-related psychiatric disorders among refugees. We found no statistically significant group differences on the primary or secondary outcome measures except a significant difference on the SDS (level of functioning) and on the WHO-5 (quality of life), but the latter only marginally significant when post-treatment scores were adjusted in relation to pre-treatment scores. In contrast to our hypothesis, the detected differences were in favour of sertraline. The relatively large number of outcome measures in this study implies a risk of random findings of significant group differences. This will statistically be the case for 5 % of the findings when a significance level of *p* = 0.05 is applied and may be the case for the few significant findings in our study. However, in spite of the few significant group differences, we found, throughout the ratings, a fairly consistent tendency to somewhat better outcome in the sertraline group, which makes it less likely that the findings are random.

The most recent meta-analysis on mixed groups of PTSD patients found the evidence for the effect of venlafaxine to be superior to that of sertraline [35]. Similarly, a meta-analysis from 2012 found a larger effect of venlafaxine on the Clinician-Administered PTSD Scale (CAPS) [36]. The fact that our study was not able to replicate this finding could be due to a range of factors. One likely explanation is the relatively low mean dose of venlafaxine that the patients received in the present study. Venlafaxine’s effect on noradrenaline reuptake is dose-dependent, starting on doses of around 225 mg daily. When the daily dose is lower, it acts on serotonin reuptake only, like a common SSRI [36, 37]. Other studies have furthermore suggested that there are biological differences between Caucasians and people from the Middle Eastern areas, including the percentage of fast metabolisers being substantially larger among Middle Eastern patients [8]. It is, however, also a possibility that the true differences between the effectiveness of sertraline and venlafaxine in treating PTSD are rather small as another meta-analysis from 2013 found only little difference in effect sizes [4].

During the study, changes in the patients’ physical condition were recorded at each medical doctor session, but only unexpected or serious events/side effects were systematically collected and reported. Therefore, we cannot for certain conclude if side effects occurred more frequently in the venlafaxine group than in the sertraline group. However, the fact that we ended up with a relatively low dose of venlafaxine, even though we aimed for maximum doses, might suggest that the acceptability of sertraline was larger than that of venlafaxine in the population of the present study. This was also the case in the only other RCT comparing sertraline and venlafaxine in trauma-affected refugees [19], in which the number of drop outs were higher in the venlafaxine group and in line with findings from studies with other psychiatric patient groups [38].

Overall we found some small but significant differences between pre-treatment and post-treatment ratings in both groups on the primary and most of the secondary outcome measures. As the patient sample in the present study compromised severely trauma-affected refugees who were referred to a specialised clinic, the rather small improvements are not surprising. Furthermore, as the pre- to post-treatment improvements presented are means of the 195 patients included in the analyses, some patients will definitely get a much larger improvement. Although out of scope of the present paper, predictors of positive treatment outcomes are important to examine in order to further improve the treatment. Using the present sample, these predictors have been analysed in a recently published paper [39].

The study design, with no placebo or control group, does not allow us to conclude whether the improvements detected are due to an effect of the treatment programme provided. However, a previous study conducted at CTP found a small but significant effect of sertraline on depression compared to waitlist controls, and it therefore seems likely that at least part of the change is due to the pharmacological treatment in the present study too [14].

As a high percentage of patients in both groups were taking a small dose of mianserin during parts of the study, the sleep enhancing effect of mianserin might contribute to the overall change. If we assume that the change we found between pre-treatment and post-treatment ratings on the majority of measures is partly due to the pharmacological treatment, it might very well be the effect of the combination of mianserin and the investigated antidepressants rather than sertraline or venlafaxine alone.

Around 21 % of the total study population took antipsychotic medication at some point during the trial. Although there was no significant group difference in the use of antipsychotics, one cannot rule out that a possible effect of antipsychotics on the patients’ PTSD might have hidden differences in effects between the two groups due to a ceiling effect on brain receptors. The general small pre-to post-treatment changes in both groups do, however, make this less likely.

The duration of the study was six months, during which the patients gradually started pharmacological treatment. This meant that patients usually received antidepressants for substantially shorter time when evaluated. It is therefore possible that part of the treatment effect only can be observed after a longer post-treatment period. For this reason, patients in the present study will be invited to follow-up interviews and rating-completion 6 and 18 months after termination of treatment programme.

### Limitations and strengths

Our trial has certain limitations. Blinded observer ratings were carried out, but neither patients nor clinical staff members were blinded to treatment allocations. This was decided by an expert team of doctors at CTP having a substantial amount of experience with, and knowledge about, trauma-affected refugees. Due to the nature of the trauma previously experienced by many of the patients (torture experiences) and the subsequent complex PTSD with suspicion as a key feature, the clinicians found it likely that a majority of the patients would decline participation if blinded. A substantial selection bias would have been very unfortunate in this study since we were aiming to get a population and a set-up that were to a large extent, comparable with any other clinic in the field, making the study results easy to implement elsewhere. In the current study design, where most outcome measures were self-report ratings, the gains of blinding would have been limited. In combination with the risk of greater selection bias, this was the basis for the current pragmatic design where blinded observer ratings were chosen as recommended by authors of similar studies in the field [19].

The study did not include a waitlist or placebo control group due to both pragmatic and ethical considerations. The primary aim of the present study was to compare the effect of the two types of antidepressants, not to measure the overall effect of the treatment programme. Since another study at the clinic, which included a waitlist control group, had recently been completed [14], we found it less important to include a waitlist control group in the present study and found it more important to achieve larger samples in the pharmacological treatment groups. However, in light of the relatively small effect sizes found in the waitlist control group trial [14] and the little evidence of differential effects of the types of pharmacological treatment, it would have been advantageous being able to compare the effects of the two pharmacological treatments with a waitlist control group. Since there was not a natural waiting list at the time of the present study it might however, have been ethically questionable to prevent participants in the study from immediate treatment.

Pill count was used to determine compliance. This is naturally a less secure method than blood tests as patients could potentially hide poor compliance from the clinical staff by throwing away their medication. However, we chose the present method based on the assumption that some patients would find the frequent collection of blood samples unacceptable due to their trauma history. Blood tests as compliance measures do however hold some advantages over pill counts. They are an objective measure of whether the patients are taking the medication and also provide some indication of whether the blood level of the medication is within a therapeutic range. This is especially relevant in non-western populations where percentages of fast drug metabolisers have been found to differ from western populations. In the present study, this difference could potentially have influenced the efficacy of the pharmacological agents investigated, and it would therefore have been beneficial to know whether blood levels of the antidepressant agents were within the therapeutic range.

Questions of validity and reliability of the ratings are important when it comes to measuring treatment outcomes in refugee populations with diverse cultural and linguistic backgrounds. While used in previous studies, the included ratings had not been validated specifically for our population, which is a general problem in refugee health research. Hollifield et al. reviewed a range of rating scales used in refugee health studies, some of which were also used in the present study. They found the HTQ and HSCL-25 to be fairly well-validated, but found the majority of the remaining reviewed ratings scales to be poorly validated for refugee groups [9]. However, in the present study, we found that Cronbach’s alpha was high for the majority of the rating scales.

The study also holds important strengths. Unlike most studies in the field, participants were randomised, which reduces selection bias and improves comparability between the two intervention groups. Furthermore, we aimed to avoid restricting the inclusion unnecessarily in order to make the patient group similar to the population treated at other refugee health care facilities both in Denmark and worldwide: refugee patients with substantial comorbidity and multiple trauma-related disorders. Notably, they are not “pure” PTSD patients and results should be interpreted in light of this fact.

The study is a pragmatic trial, which means that we investigated the drugs as they are used in a real life setting. The disadvantage is that patients do not always follow clinicians’ advice and guidelines, resulting in non-optimal dosages of the investigated drugs and discontinuation of treatment for a variety of reasons. Consequently, the results in our study contribute to our knowledge on the effects of sertraline and venlafaxine in the dosages that has been possible to achieve for typical patients in a refugee healthcare setting – not the drugs taken in maximum dosages in a controlled setting with a carefully selected group of patients. An important advantage of this approach is that the results and lessons learned from this study are, to a large extent, transferrable to similar refugee health facilities. The design is fairly simple and manuals were used for all interventions, which makes the study easy to replicate in similar or larger settings.

## Conclusion

In the present study, sertraline had a somewhat better outcome than venlafaxine on several secondary outcome measures, although no difference was found on the primary outcome measure, the HTQ. As mentioned above, this is in line with the findings of a similar study conducted previously [19]. Although differences in the effects of the two drugs were small and possibly due to the low dosage of venlafaxine achieved, sertraline used in a pragmatic clinical setting seems to be at least as good as venlafaxine and possibly better tolerated. Nonetheless, a final conclusion on tolerability cannot be drawn based on the data of the presented study alone.

The present study has brought new knowledge on the effects of sertraline and venlafaxine in trauma-affected refugees. Still, the drugs investigated are merely two out of a range of psychotropis used in refugee mental health settings. Hence, there is still an urgent need for large-scale randomised studies of both trauma-affected refugees and other PTSD populations in order to determine the efficacy of other pharmacological agents typically used in the field, for example TCAs, newer antidepressants such as agomelatine, and other types of psychotropics such as topiramate or prazozin.

## Additional file

Additional file 1: Table S1. Score differences between pre-treatment and post-treatment ratings (rating completers). Overview over the pre-treatment and post-treatment rating scores rating for all patients who have completed a both pre-and post-treatment ratings. (DOCX 23 kb)

## Abbreviations

ACT: Acceptance and commitment therapy
CAPS: Clinician-administered PTSD scale
CBT: Cognitive behavioural therapy
CSS: Crisis support scale
CTP: Competence centre for transcultural psychiatry
GAF: Global assessment of functioning
HAM-A: Hamilton anxiety scale
HAM-D: Hamilton depression scale
HSCL-25: Hopkins symptom checklist-25
HTQ: Harvard trauma questionnaire
MTV: Abbreviation for medical technology assessments in Danish. Until 2012 this was the name of the Danish health authorities’ official assessments of evidence for interventions used within the health sector
PTSD: Post-traumatic stress disorder
RCT: Randomised controlled trial
SAS-SR: Social adjustment scale - self report
SCL-90: Symptom checklist-90
SDS: Sheehan disability scale
SNRI: Serotonin–norepinephrine reuptake inhibitors
SSRI: Selective serotonin reuptake inhibitors
TF-CBT: Trauma-focused cognitive behavioural therapy
VAS: Visual analogue scale
WHO-5: WHO-five well-being index
