# Supplementary material for: Treatment of trauma-affected refugees with venlafaxine versus sertraline combined with psychotherapy - a randomised study
Source: BMC Psychiatry. 2016 Nov 8;16:383. doi: 10.1186/s12888-016-1081-5 (PMC5101827; doi:10.1186/s12888-016-1081-5)
Supplement: Additional file 1: Table S1. — Score differences between pre-treatment and post-treatment ratings (rating completers). Overview over the pre-treatment and post-treatment rating scores rating for all patients who have completed a both pre-and post-treatment ratings. (DOCX 23 kb) [file 12888_2016_1081_MOESM1_ESM.docx]

Additionsl file 1

**Table S1. Score differences between pre-treatment and post-treatment ratings (rating completers)**

| **Rating** | **Group (n)** | **Mean pre-treatment score (SE)** | **Mean post-treatment score (SE)** | **Difference (SE)** | **P-value** |
| --- | --- | --- | --- | --- | --- |
| HTQ | Sertraline (85) | 3.24 (0.04) | 3.02 (0.06) | **0.22 (0.06)** | <0.01** |
|  | Venlafaxine (69) | 3.21 (0.05) | 3.06 (0.07) | **0.15 (0.06)** | 0.01** |
|  | Difference | 0.03 (0.06) | -0.04 (0.09) | 0.07 (0.08) | 0.33 |
| HSCL-25 | Sertraline (84) | 3.02 (0.0.05) | 2.84 (0.07) | **0.18 (0.07)** | 0.01** |
|  | Venlafaxine (66) | 3.09 (0.0.05) | 2.96 (0.07) | **0.13 (0.05)** | 0.02* |
|  | Difference | -0.07 (0.07) | -0.12 (0.10) | 0.05 (0.08) | 0.59 |
| SCL-90 | Sertraline (81) | 2.43 (0.09) | 2.37 (0.10) | **0.06 (0.10)** | 0.58 |
|  | Venlafaxine (66) | 2.60 (0.10) | 2.58 (0.09) | **0.02 (0.07)** | 0.79 |
|  | Difference | -0.17 (0.13) | -0.21 (0.14) | 0.04 (0.13) | 0.76 |
| VAS | Sertraline (79) | 6.50 (0.24) | 6.22 (0.29) | **0.28 (0.26)** | 0.27 |
|  | Venlafaxine (64) | 6.79 (0.27) | 6.81 (0.25) | *-0.02 (0.21)* | 0.92 |
|  | Difference | -0.28 (0.26) | -0.59 (0.39) | 0.26 (0.34) | 0.36 |

***Symptoms self-ratings***

HTQ, HSCL-25, SCL = 1-4 (1 best score), VAS = 0-10 (0 best score)

HTQ: Harvard Trauma Questionnaire

HSCL-25: Hopkins Symptom Checklist-25

SCL-90: Symptom Checklist-90

VAS: Visual Analogue Scale (for pain)

***Life quality/level of functioning self-ratings***

| **Rating** | **Group (n)** | **Mean pre-treatment score(SD)** | **Mean post-treatment score (SD)** | **Difference (SD)** | **P-value** |
| --- | --- | --- | --- | --- | --- |
| WHO-5 | Sertraline (85) | 13.04 (1.52) | 22.33 (2.71) | **-9.29 (2.42)** | <0.01** |
|  | Venlafaxine (67) | 14.51 (1.88) | 17.85 (2.34) | **-3.34 (2.20)** | 0.14 |
|  | Difference | -1.47 (2.42) | 4.48 (3.58) | 5.95 (3.28) | 0.07 |
| SDS | Sertraline (80) | 24.54 (0.60) | 21.71 (0.91) | **2.84 (0.87)** | <0.01** |
|  | Venlafaxine (67) | 22.52 (0.74) | 23.16 (0.79) | *-0.63 (0.89)* | 0.48 |
|  | Difference | 2.02 (0.94)* | -1.45 (1.21) | 3.47 (1.24) | <0.01** |
| SAS-SR | Sertraline (86) | 3.00 (0.08) | 2.72 (0.08) | **0.28 (0.07)** | <0.01** |
|  | Venlafaxine (71) | 3.00 (0.08) | 2.84 (0.08) | **0.16 (0.08)** | 0.03* |
|  | Difference | 0.01 (0.11) | -0.12 (0.11) | 0.11 (0.11) | 0.31 |
| CSS | Sertraline (86) | 21.91 (0.93) | 22.64 (0.81) | **-0.73 (0.80)** | 0.36 |
|  | Venlafaxine (72) | 22.28 (0.85) | 22.33 (0.80) | **-0.05 (0.74)** | 0.95 |
|  | Difference | -0.37 (1.26) | 0.31 (1.14) | -0.68 (1.09) | 0.53 |

WHO-5 = 0-100 (100 best score), SDS = 0-10 (0 best score), SAS-SR = 1-5 (1 best score), CSS = 1-7 (7 best)

WHO-5: WHO-Five Well-being Index

SDS: Sheehan Disability scale

SAS-SR: Social Adjustment Scale - Self Report

CSS: Crisis Support Scale

***Observer ratings***

| **Rating** | **Group (n)** | **Mean pre-treatment score(SD)** | **Mean post-treatment score (SD)** | **Difference (SD)** | **P-value** |
| --- | --- | --- | --- | --- | --- |
| HAM-D | Sertraline (89) | 23.84 (0.58) | 22.29 (0.86) | **1.55 (0.79)** | 0.05* |
|  | Venlafaxine (69) | 23.61 (0.65) | 22.52 (0.92) | **1.09 (0.82)** | 0.19 |
|  | Difference | 0.23 (0.88) | -0.23 (1.26) | 0.46 | 0.69 |
| HAM-A | Sertraline (88) | 26.84 (0.72) | 26.31 (1.04) | **0.53 (0.97)** | 0.58 |
|  | Venlafaxine (69) | 27.09 (0.74) | 26.23 (1.07) | **0.86 (0.99)** | 0.39 |
|  | Difference | -0.25 (1.03) | 0.08 (1.49) | -0.32 (1.39) | 0.82 |
| GAF-S | Sertraline (68) | 47.50 (0.69) | 51.37 (0.99) | **3.87 (0.77)** | <0.01** |
|  | Venlafaxine (56) | 48.16 (0.71) | 51.84 (0.95) | **3.68 (1.08)** | <0.01** |
|  | Difference | -0.66 (0.99) | -0.47 (1.37) | 0.19 (1.33) | 0.89 |
| GAF-F | Sertraline (67) | 48.55 (0.82) | 50.27 (1.00) | **1.72 (0.80)** | 0.03* |
|  | Venlafaxine (56) | 48.75 (0.72) | 51.77 (1.00) | **3.02 (0.90)** | <0.01** |
|  | Difference | -0.20 (1.09) | -1.50 (1.41) | -1.30 (1.20) | 0.28 |

HAM = 0-4 (0 best score), GAF = 0-100 (100 best score)

HAM-A: Hamilton Anxiety scale and HAM-D: Hamilton Depression scale

GAF: Global Assessment of Functioning (S=symptom score and F= functioning score)

*Overview over the pre-treatment and post-treatment rating scores rating for all patients who have completed a both pre-and post-treatment ratings. Numbers in brackets after group indicates the number of patients included in this analysis. In the right column the p-values refer to the significance of differences between pre- and post-treatment ratings in each group and the significance of group differences in the difference between pre- and post-treatment ratings (corresponding to the interaction between intervention group and rating time).*

*** =** statistically significant (p= 0.05 or below)

** = higly statistical significant (p = 0.01 or below)

**Bold =** Improvement

*Italic* = Deterioration
